# Towards Understanding the Development of Breast Cancer: The Role of RhoJ in the Obesity Microenvironment

**DOI:** 10.3390/cells13020174

**Published:** 2024-01-17

**Authors:** Lara J. Bou Malhab, Vidhya A. Nair, Rizwan Qaisar, Gianfranco Pintus, Wael M. Abdel-Rahman

**Affiliations:** 1 Research Institute of Medical and Health Sciences, University of Sharjah, Sharjah 27272, United Arab Emirates; vnair@sharjah.ac.ae; 2 Basic Medical Sciences, College of Medicine, University of Sharjah, Sharjah 27272, United Arab Emirates; rqaisar@sharjah.ac.ae; 3 Department of Biomedical Sciences, University of Sassari, 07100 Sassari, Italy; gpintus@uniss.it; 4 Department of Medical Laboratory Sciences, College of Health Sciences, University of Sharjah, Sharjah 27272, United Arab Emirates

**Keywords:** breast cancer, chronic inflammation, obesity, RhoJ, tumor micro-environment

## Abstract

Obesity is a growing pandemic with an increasing risk of inducing different cancer types, including breast cancer. Adipose tissue is proposed to be a major player in the initiation and progression of breast cancer in obese people. However, the mechanistic link between adipogenicity and tumorigenicity in breast tissues is poorly understood. We used in vitro and in vivo approaches to investigate the mechanistic relationship between obesity and the onset and progression of breast cancer. In obesity, adipose tissue expansion and remodeling are associated with increased inflammatory mediator’s release and anti-inflammatory mediators’ reduction.. In order to mimic the obesity micro-environment, we cultured cells in an enriched pro-inflammatory cytokine medium to which we added a low concentration of beneficial adipokines. Epithelial cells exposed to the obesity micro-environment were phenotypically transformed into mesenchymal-like cells, characterized by an increase in different mesenchymal markers and the acquisition of the major hallmarks of cancerous cells; these include sustained DNA damage, the activation of the ATR-Chk2 pathway, an increase in proliferation rate, cell invasion, and resistance to conventional chemotherapy. Transcriptomic analysis revealed that several genes, including RhoJ, CCL7, and MMP9, acted as potential major players in the observed phenomenon. The transcriptomics findings were confirmed in vitro using qRT-PCR and in vivo using high-fat-diet-fed mice. Our data suggests RhoJ as a potential novel molecular driver of tumor development in breast tissues and a mediator of cell resistance to conventional chemotherapy through PAK1 activation. These data propose that RhoJ is a potential target for therapeutic interventions in obese breast cancer patients.

## 1. Introduction

Obesity has become a worldwide concern because of its impact on public health. By 2025, global obesity prevalence is expected to reach 18% in men and more than 21% in women [1]. Obesity is an excess of body fat, considered to be harmful to health and defined by the World Health Organization (WHO) as a body mass index (BMI) (body weight in kilograms (Kg) divided by height in meters squared (m^2^)) of >30 kg/m^2^ (class 1: 30–35, class 2: 35–40, and class 3: >40), with overweight values at 25–29.9 kg/m^2^ and normal values considered 18.5–24.9 kg/m^2^ [2]. The increased prevalence of obesity is influenced by multifactorial conditions, but the main driver is believed to be an overall increase in caloric intake [3] combined with a sedentary lifestyle [4]. Obesity is an established risk factor for the development of multiple cancer types, including carcinomas of the breast, colon, endometrium, esophagus, gall bladder, and kidney [5,6].

Breast cancer is the most common cancer among women worldwide, with nearly 672,000 deaths registered in 2018 [7]. During breast carcinogenesis, mammary epithelial cells undergo a series of molecular and morphological changes, resulting in their transformation into cancer cells. This phenomenon is reinforced by the composition of breasts (they comprise three major components: fibrous tissue, glandular tissue, and adipose tissue) and the interaction between these different compartments [8]. In obese people, excess energy is stored as triglycerides in adipocytes, leading to their modification and expansion through both hypertrophy and hyperplasia [9]. Once adipocytes undergo hypertrophy, they endure mechanical stress from interaction with neighboring cells and extracellular matrix [10], resulting in dysfunctional adipose tissue, marked by the elevated secretion of adipokines and pro-inflammatory cytokines, which stimulate low-grade pathological inflammation in the adipose tissue [11]. In this regard, the over-secretion of numerous pro-inflammatory adipokines, such as leptin, tumor necrosis factor-alpha (TNF-α), plasminogen activator inhibitor-1 (PAI-1), interleukin-8 (IL-8), hepatocyte growth factor (HGF), visfatin, and resistin, combined with a decrease in beneficial adipokine secretion, including adiponectin and omentin, have been reported in previous studies [12,13,14,15,16].

According to the literature, in several breast cancer cases, the connective tissue separating adipocytes from tumor cells is reduced, facilitating tumor cells to break through the basement membrane. This results in the juxtaposition of adipocytes and breast cancer cells, allowing their interaction [17]. It was shown that MCF-7 cell exposure to adipocyte-secreted factors stimulated their migration and proliferation potential. In vivo, tumors formed from SUM-159PT human breast adenocarcinoma cells co-injected with murine adipocytes showed a significant increase in the size of the formed tumors when compared to SUM-159PT cells co-injected with murine fibroblasts [18]. Micro-array analysis performed on MCF-7 breast cancer cells treated with conditioned media formed from either murine adipocytes or fibroblasts revealed that adipocyte-conditioned media upregulated the genes involved in proliferation, invasion, and metastasis [18]. In addition, leptin, a product of the Ob gene that is secreted by adipocytes, was found to be overexpressed in nearly 92% of breast carcinomas. Interestingly, 31% of the cases showed the presence of a distant metastasis accompanied by leptin overexpression [19]. The inflammatory cytokine TNF-α was the first cytokine identified as being secreted by adipocytes [20]. In obese individuals, an increase in circulating TNF-α due to an overall increase in fat tissue could lead to breast cancer tumorigenesis by contributing to insulin resistance and IL-6 synthesis regulation, considering that TNF-α was identified as a key regulator of IL-6 synthesis [21]. IL-6 is expressed and secreted by adipocytes. Its expression is proportional to BMI, where IL-6 expression is found to be elevated in obese individuals [22]. Interestingly, it was shown that IL-6 is able to promote cell migration through MAPK activation.

The key regulator of adipogenesis was found to be dysregulated in obese people, leading to an increase in the production of the plasminogen-activator inhibitor-1 (PLAI-1). PLAI-1 upregulation was associated with angiogenesis, the enhancement of cell adhesion, migration, and the inhibition of apoptosis, and it is suspected to be responsible for the breast micro-environment facilitating cancer development and metastasis [23,24,25].

In obesity, breast adipose tissue undergoes significant changes in the secretion of both hormonal and inflammatory molecules, creating a mitogenic micro-environment [26]. Given the crosstalk between breast epithelial cells and the adipose tissue, it is plausible to view this interaction as a significant player in the initiation and progression of breast cancer in obese individuals [27]. In a study conducted by Vona-Davis et al., adipokines were a major factor contributing to obesity-associated breast cancer [28,29]. Moreover, in a meta-analysis study, low circulating adiponectin levels were associated with increased breast cancer risk [30,31].

Rho GTPases are small guanine nucleotide-binding proteins that are known to be critical regulators of the actin cytoskeletal structure and the dynamics that are required for cellular migration [32]. Three prototypical members of this family, including RhoA, Rac1, and CDC42, have critical roles in lamellipodia, stress fiber, and filipodia formation [33]. RhoJ, also known as TCL (TC10-like), is a recently identified member of the CDC42 subfamily GTPases [34]. Despite sharing structural similarities and common effector molecules with other Rho GTPases, RhoJ has its distinct characteristics. RhoJ regulates cellular migration and invasion by altering actin cytoskeletal dynamics [35] and is involved in the early stages of adipocyte differentiation [36]. All Rho GTPases can shuttle between the GTP-bound active form and GDP-bound inactive form. The transition between active and inactive forms is regulated by guanine nucleotide exchange factors (GEFs) and GTPase-activating proteins (GAPs). Guanine nucleotide dissociation inhibitors (GDIs) are known to bind the GDP form of Rho GTPases and sequester them in the cytosol under their inactive form [33].

To date, direct mechanistic evidence that the altered obesity micro-environment can induce mammary epithelium transformation is still lacking. We aimed to address this research gap by investigating the transformation-associated morphological and molecular changes of mammary epithelial cells exposed to a mimic of an obesity micro-environment. By using a dual approach with in vitro cell culture and an in vivo mouse model of obesity, we identified the Rho GTPase RhoJ as a major candidate driving breast cancer in an obesity environment. Other potential molecules (CCR5, CCL7, PAK1, and MMP9) were identified as potential RhoJ effectors, ensuring cancer cells resistance to conventional chemotherapy and invasiveness and maintaining an inflammatory micro-environment.

## 2. Materials and Methods

### 2.1. Chemicals

The chemicals tested were selected based on the micro-environment generated in obese people, which is enriched with inflammatory mediators, cytokines, and low concentrations of adiponectin. These include leptin, Adiponectin, IL-6, TNF-a, and PAI-1. A conditioned media protocol was adapted from [37]. The different chemicals were purchased from Sigma-Aldrich (St. Louis, MO, USA). The chemicals were dissolved, as recommended, either with 100% dimethyl sulfoxide, nuclease-free water, or 1% BSA and stored as stock solutions at −20 °C or −80 °C. doxorubicin and hydrogen peroxide were purchased from Sigma-Aldrich (St. Louis, MO, USA). Trypsin was purchased from Sigma-Aldrich (St. Louis, MO, USA).

### 2.2. Cell Lines and Cell Culture 

MCF7 breast cancer cells (estrogen receptor (ER)-positive, progesterone receptor (PR)-positive, human epidermal growth factor receptor 2 (HER2)-negative, and luminal A), MDA-MB-231 (ER-negative, PR-negative, HER2-negative, and triple-negative), MDA-MB-361 (ER-positive, PR-negative/positive, HER2-positive, and luminal B), BT549 (ER-negative, PR-negative, HER2-negative, and triple-negative), and ZR751 (ER-positive, PR-positive/negative, HER2-negative, and luminal A) [38] were cultured in Dulbecco’s modified Eagle’s Medium Sigma-Aldrich (St. Louis, MO, USA), and immortalized normal epithelial cells HME1 (hTERT-HME1) were cultured in Dulbecco’s modified Eagle’s Medium F-12 (DMEM F-12) (Sigma-Aldrich, St. Louis, MO, USA) with 50 U/mL Penicillin, 50 μg/mL Streptomycin, and 10% FCS (fetal calf serum) at 37 °C, 5% CO_2_. The cells were split according to their appropriate splitting ratio when the confluency reached 80–90%, as instructed by the supplier. 

HME1 cells were cultured in Dulbecco’s modified Eagle’s Medium F-12 (Sigma-Aldrich, St. Louis, MO, USA) to which we added the different compounds at their appropriate concentration: leptin (1000 ng/mL) [39], adiponectin (50 ng/mL), IL-6 (50 ng/mL) [40,41], TNF-α (50 ng/mL) [42], and PAI-1 (20 ng/mL) [43]. The cells were split when confluency had reached 80%, and the different molecules were added to the medium. This was repeated twice a week for 2 months in a row.

To induce the differentiation of 3T3-F442A cells, the cells were grown in DMEM and 10% FCS with 50 U/mL penicillin and 50 μg/mL streptomycin. Cells will differentiate into adipocytes once confluent, which takes approximately 10 days until confluency (where they became differentiated into adipocytes). Cells became rounded and enlarged with lipid droplet accumulation in their cytoplasm. For differentiation verification, the cells were fixed with 10% (*v*/*v*) formalin in phosphate-buffered saline and then stained with Oil Red O, as described by Kuri-Harcuch and Green (1978) [44]. The cells were considered lipid-positive when the droplets were stained red.

The CM was collected, centrifuged at 300 g for 5 min, filtered through a 0.2 μm filter, and stored at −20 °C before use.

### 2.3. Cell Lysis and Protein Quantification

Cells were rinsed twice with cold PBS (Sigma-Aldrich, St. Louis, MO, USA) and then subjected to trypsin for 5 min at 37 °C. The cells were then collected with their appropriate media and centrifuged at 1100 rpm for 5 min. The supernatant was discarded, and the pellet was washed twice with cold, 1X PBS, and then re-suspended in 50–1000 µL of 2x Laemmeli Buffer. Bradford protein assay (Bio-Rad, Hercules, CA, USA) was performed to quantify protein concentration. The cell lysate was stored at −20 °C.

### 2.4. Western Blot Analysis

The protein lysates acquired from the cells were loaded into SDS-PAGE (20 µg per well) along with molecular weight marker (BLUltra Prestained Protein Ladder) (BIO-HELIX, New Taipei City, Taiwan). The gel with separated proteins was transferred onto (PVDF) polyvinylidene difluoride membrane (Bio-Rad, Hercules, CA, USA). Membranes were blocked with 5% BSA solution (Sigma-Aldrich, St. Louis, MO, USA) (TBS with 5% BSA and 0.1% Tween-20) for 1 h at room temperature. The primary antibodies diluted in TBS 0.1% Tween-20 with 5% BSA were incubated with the membranes overnight at 4 °C. Membranes were washed three times with TBS 0.1% Tween-20 (10 min each) and then incubated with the secondary antibodies (Mouse or Rabbit) (Sigma-Aldrich, St. Louis, MO, USA) diluted 1:2000 in 5% BSA at room temperature for 1 h. Membranes were washed three times with TBS 0.1% tween-20 for 10 min each. Membranes were incubated with HRP-labeled substrate (Pierce™ ECL Western Blotting Substrate) (Thermo Scientific, Rockford, IL, USA) for 2 min and exposed to medical film (CL-XPosureTM Film) (Thermo scientific, Rockford, IL, USA). The table below summarizes the different antibodies used (Table 1).

### 2.5. Gene Expression Analysis Using qRT-PCR

#### 2.5.1. RNA Extraction

mRNA was extracted using RNAeasy Mini Kit (Qiagen, Hilden, Germany). Cells were harvested when they reached 80% confluency. Cells were washed twice with warm PBS and trypsinized, then centrifuged for 5 min at 1100 rpm. The pellet was resuspended in 350 µL of RLT buffer. The same volume of 70% ethanol was used. The total volume was transferred to an RNase spin column placed in a 2 mL collecting tube. The column was centrifuged for 15 s at >10,000 rpm at room temperature, the flow through was discarded, and different buffers were added: 700 µL of washing buffer (RW1), followed by 500 µL of RPE buffer, every time the column was centrifuged for 15 s at >10,000 rpm at room temperature. A total of 500 µL of RPE buffer was added, and the column was centrifuged at 11,000 rpm for 2 min at 23 °C. The total mRNA was collected by adding 30–50 µL of RNase free to the column membrane and then centrifuged for 1 min at 11,000 rpm. The RNA concentration was measured by Nanodrop (Colibri Microvolume Spectrometer from Titertek-Berthold) by direct absorbance at A280. The collected RNA extraction was kept for long storage at −80 °C.

#### 2.5.2. Reverse Transcription

cDNA was synthetized using QuantiTect Reverse Transcription Kit (Qiagen, Hilden, Germany). A total of 1 µg of RNA was mixed with 2 μL of gDNA wipeout buffer, Rnase-free water was added to a final volume of 14 μL. Then, the samples were heated for 2 min at 42 °C. A total of 1 μL of Quantiscript Reverse transcriptase, 1 μL RT primer mix, and 4 μL Quantiscript RT Buffer were added to each of the resultant samples from the previous step. The samples were heated for 15 min at 42 °C and then 3 min at 95 °C. cDNA was kept at −20 °C for storage.

#### 2.5.3. qRT-PCR

qRT-PCR was performed using 5x HOT FIREPol^®^ EvaGreen^®^ qPCR Mix Plus (ROX) (Solis BioDyne, Tartu, Estonia). mRNA expression levels were amplified using the QuantStudio3 system by loading 50 ng of cDNA per well, mixed with 1x HOT FIREPol EvaGreen qPCR Mix and 0.2 µM of the corresponding primer. GAPDH and RPL18 were used as an internal control, and 2^−ΔΔct^ was used to calculate mRNA relative expression. The below table summarizes the different primers used and their sequences (Table 2).

### 2.6. RNA Sequencing

Total mRNA was isolated from both cells (HME1 and HME1) (AK + I) and then submitted to MedGenome Labs Pvt Ltd., Bangalore, India, by the local dealer: Genetrics, Dubai, UAE. RNA samples were quantified using Qubit RNA Assay HS (Invitrogen, Cat# Q32852). RNA purity was checked using QIAxpert, and RNA integrity was assessed on TapeStation using RNA ScreenTapes (Agilent Technologies, Santa Clara, CA, USA, Cat# 5067-5579). All RNA samples passed the QC and were passed for the RNA library prep.

#### 2.6.1. RNA Library Prep Protocol

NEB Next Ultra RNA Library prep kit (NEB, Ipswich, MA, USA, #E7530L) was used to prepare the libraries for RNA sequencing. First, mRNA was selected using Poly-A selection kit (Lexogen, Vienna, Austria, # K39.100) from Lexogen. Following purification, the mRNA was fragmented using divalent cations under elevated temperatures. The cleaved RNA fragments were copied into first-strand cDNA using reverse transcriptase. Second-strand cDNA synthesis was performed using DNA Polymerase I and RNase H enzyme. The cDNA fragments were then subjected to a series of enzymatic steps that repair the ends, tailing the 3′ end with a single ‘A’ base, followed by the ligation of the adapters. The adapter-ligated products were then purified and enriched using the following thermal conditions: initial denaturation 98 °C for 30 s; 12 cycles of −98 °C for 10 s and 65 °C for 75 s; final extension of 65 °C for 5 min. PCR products are then purified and checked for fragment size distribution on TapeStation using D1000 DNA ScreenTapes (Agilent Technologies, Santa Clara, CA, USA, Cat# 5067-5582).

#### 2.6.2. Sequencing Protocol

Prepared libraries were quantified using Qubit High Sensitivity Assay (Invitrogen, Cat# Q32852). The obtained libraries were pooled and diluted to a final optimal loading concentration before cluster amplification on an Illumina flow cell. Once the cluster generation is completed, the cluster flow cell is loaded into an Illumina HiSeq 4000 instrument to generate 60 M, 100 bp paired-end reads. More than 2000 dysregulated genes were selected and classified into two groups: upregulated and downregulated genes. This list of genes was filtered twice, keeping only the significant genes with a *p* value of <0.05 and a fold change of greater than 1.8.

These different genes were then classified into pathways by using Panther version 16.0, and the gene panel selected for our study was based on the pathways involved in chronic inflammation and carcinogenesis with the most significant fold change.

### 2.7. Invasion Assay

A QCM TM High-Sensitivity Non-cross-linked Collagen Invasion Assay kit was used for invasion assays (Millipore, MA, USA). Modified chambers with filter inserts (pore size: 8 μm) coated with Matrigel in 24-well dishes were used. A total of 300 µL of serum-free media was added per chamber for 30 min at room temperature. A total of 250 μL of the media were removed from the inserts. About 0.5 million cells in serum-free media were added into the 250 μL serum-free media per chamber (top chamber), and 500 μL of 10% FBS-containing media was placed in the bottom chambers. After 24 h of incubation, the remaining cells in the top chamber were removed with a pipette, and the cells were stained by adding 400 μL of cell stain for 15 min. After several washes with distilled water, the inserts were dried and viewed under the microscope to monitor their morphology and abundance. Then, 200 μL of extraction buffer was added and incubated for 15 min at room temperature. The dye mixture was then assessed by a plate reader at 492 nm and 630 nm.

### 2.8. Annexin V-FITC/PI Staining

An annexin V-FITC/PI staining kit was obtained from BD Biosciences (San Jose, CA, USA). HME1 and HME1 (AK + I) were grown in 25 cm^2^ cell culture dishes for 24 h and used for annexin V-FITC/PI experiments. Briefly, the cells were trypsinized, washed twice with PBS, and collected at a concentration of 1 × 10^5^ cells/mL in 1X binding buffer. Cells were then incubated with 5 μL of FITC annexin V and 5 μL PI for 15 min at room temperature in the dark. Cells were analyzed with a BD FACS Aria III (BD Biosciences, San Jose, CA, USA) using excitation/emission wavelengths of 488/525 nm and 488/675 nm for annexin V and PI, respectively. Live, apoptotic, and necrotic cells were determined by calculating the sum of the percentage of cells present in the annexin V +ve/PI –ve and annexin V +ve/PI +ve quadrants.

### 2.9. Soft Agar Colony Formation

A total of 5 × 10^3^ of HME1, HME1 (AK + I), and Hela cells were mixed with 0.6% agarose (mixed with the adequate medium *v*:*v*) and were plated over a 1% agarose layer (mixed with the adequate medium *v*:*v*) (Sigma-Aldrich, St. Louis, MO, USA). The medium was renewed twice weekly by adding 200 μL of media alone to the HME1 and Hela cells and media with the adipokine and inflammatory mediators for HME1 (AK + I). After 21–30 days, colonies had formed; they were fixed using 4% PFA, washed, and further stained with 0.05% crystal violet solution (Sigma-Aldrich, St. Louis, MO, USA). The colonies were photographed at 40× magnification using a camera fitted with an inverted microscope. Cell colonies were counted manually in four randomly selected magnification fields; the data represent the number of colonies. Three independent experiments were performed.

### 2.10. Cell Viability and Proliferation

A total of 2 × 10^3^ HME1 and HME1 (AK + I) were seeded per well in 96-well plates. After 24 h incubation in their appropriate media (DMEM-F12 or DMEM-F12 supplemented with cytokines, inflammatory mediators, and adiponectin), the cells were either treated (or not) in triplicate with doxorubicin overnight. MTT (bioWORLD, Irving, TX, USA) was added, and the cells were incubated for 2–4 h at 37 °C. Then, MTT was removed, and 100 μL of 100% DMSO was added per well. Cells were kept at room temperature, shaking for 5 min; the readings were taken at 570 nm using Corning^®^ 96-well clear flat bottom polystyrene TC-treated microplates (Costar^®^, Kennebunk, ME, USA).

### 2.11. Immunofluorescence

Cells were plated on a glass coverslip at ~25% confluence and maintained under culture conditions (described above) either with DMEM-F12 alone or with cytokines, inflammatory mediators, and adiponectin. Cells were washed twice with PBS and fixed at room temperature with 4% paraformaldehyde for 5–10 min, followed by three additional washes in ice-cold 1X PBS and permeabilized with PBS containing 0.1% triton X-100% Sigma-Aldrich (St. Louis, MO, USA). Subsequently, cells were treated with blocking buffer (1% BSA containing 0.05% Tween-20) for 30 min at room temperature, followed by overnight incubation with the primary antibody. The next day, the cells were washed three times with 1X PBS (0.05% Tween-20) for 10 min each, followed by 1 h incubation in the dark with the secondary antibody. Cells were washed three times with 1X PBS (0.05% Tween-20) for 10 min each. Coverslips were mounted on glass slides (VWR, Radnor, PA, USA). Quantification was performed by counting 100 cells per condition for three independent experiments (Table 3).

### 2.12. Animals and Diet

Female, wild-type c57BL/6j mice were maintained under specific pathogen-free conditions, housed as three mice/cage in a 12:12 (light:dark) cycle; they were provided with food and water ad libitum, as described previously [45]. From 16 weeks of age, the mice were divided into two groups, including a control group fed on a chow diet (Purina, St. Louis, MO, USA) and an experimental group fed on a 60% high-fat diet (C1090-60) (Altromin, Lage, Germany) for 14 weeks to induce obesity [46]. The mice were monitored daily for measurements of body weights and any potential signs of distress. At sacrifice, mice were euthanized via cervical dislocation, and the mammary glands were immediately excised, snap-frozen, and stored at −80 °C for further analysis. The Animal Care and Use Committee of the Sharjah Institute of Medical Research, University of Sharjah, approved the experimental protocol.

### 2.13. Protein Extraction from Mammary Gland

Mammary glands were extracted and collected in 2 mL, labeled Eppendorf tubes, and snapped frozen with liquid nitrogen. The tissue was then minced using a motor and pestle. Approximately 500 μL of RIPA lysis buffer was added per sample and kept on ice for 20 min. Samples were then sonicated for 10 s for three rounds. Samples were centrifuged for 30 min at 14,000 rpm. The fat was removed, and the proteins were collected and quantified. Protein lysates acquired from the tissues were loaded into SDS-PAGE (30 µg per well) along with molecular weight marker (BLUltra Prestained Protein Ladder) (BIO-HELIX, New Taipei City, Taiwan). 

### 2.14. Statistical Analysis 

ImageJ (1.53i 24 March 2021) was used for protein band intensity quantification. The quantification reflects the relative amounts as a ratio of each protein band relative to the lane’s loading control.

GraphPad Prism (version 9.0.0) was used for graph preparation and value analysis. A *p* value of <0.05 is represented by one star (*), *p* < 0.01 is represented by two stars (**), *p* < 0.001 is represented by three stars (***). The error bars were based on the standard deviation.

## 3. Results

### 3.1. Obesity Micro-Environment Promotes Cellular Morphology Changes, Epithelial-to-Mesenchymal Transition, and Invasion

**Figure 1:**
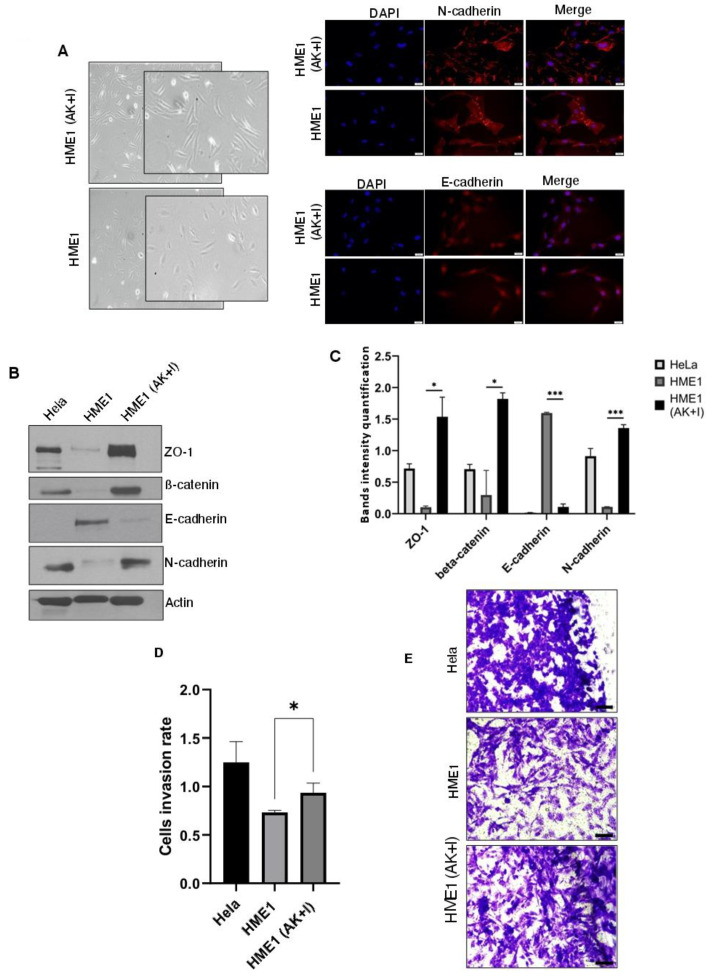
Obesity micro-environment promotes epithelial-to-mesenchymal transition, promoting cell invasion. (**A**) Epithelial cells undergo cytoskeleton changes in the obesity micro-environment. The HME1 cells were cultured in an obesity micro-environment: adipokines and inflammatory mediators are indicated as HME1 (AK + I) or HME1 when cultured in their normal media. HME1 (AK + I) acquired mesenchymal morphology. The phase-contrast images were taken at 100× magnification. HME1 and HME1 (AK + I) cells were stained for N-cadherin expression (red). Nuclei were stained with DAPI (blue). Scale bar: 100 µm. (**B**) HME1 cells lose their epithelial properties in the obesity micro-environment. Both cell types, HME1 and HME1 (AK + I), were lysed, and the extracts were used for Western blotting. Actin was used as a loading control. (**C**) EMT markers quantification. Values are represented as mean ± SEM, n = 3 (3 biological × 3 technical). Paired T test; * *p* < 0.05, *** *p* < 0.001. (**D**) HME1 cells acquired invasive properties. HME1 and HME1 (AK + I) cells were grown in a Boyden chamber with collagen-coated base–trans-well cell invasion assay, as described in Materials and Methods. Quantitative evaluation of invasive potential after dye elution and spectrophotometric reading at 560 nm. Values are represented as mean ± SEM, n = 3 (3 biological × 3 technical). Paired T test; * *p* < 0.05. (**E**) Pictures were taken under the microscope after crystal violet staining (60× magnification) to visualize the morphology of the invasive cells across the collagen-coated membrane. Scale bar: 1 mm. The highly invasive Hela cell line served as a positive control. HME1 (AK + I) exhibited a greater number of invading cells compared to HME1.

In order to establish the relationship between breast cancer and the obesity micro-environment, we first cultured human mammary epithelial (HME1) cells in their normal medium with a low concentration of adiponectin and high concentrations of leptin, IL-6, TNF-α, and PAI-1 (these cells were labeled as HME1 (AK + I) for adipokines + inflammatory mediators). The selection of adipokines and inflammatory mediators was based on the accumulated data in the literature regarding the stimulation of an obesity micro-environment. To our knowledge, a combination of the above-mentioned adipokines and inflammatory mediators has never been investigated before. We tested adipokine alone (conditioned media) as a control or in combination with inflammatory mediators. The selected dose range was guided by the literature data, with a focused dose that showed an impact on cell proliferation [47,48,49]. We ran a pilot study using cell viability, cytotoxicity, and apoptosis assays to test the impact of these conditions on the mammary epithelial cells. We selected the condition that exerted the most favorable effect on cell cytoskeleton changes. Technically, cell transformation is a long process that usually takes years. For this purpose, the HME1 cells were exposed to the obesity micro-environment conditions for two months until we started to see morphological changes [50]. The HME1 (AK + I) cells developed a mesenchymal phenotype with visible cellular elongation and protrusions (Figure 1A) compared to the HME1 cells cultured in their standard medium. The immunofluorescence images showed a clear expression of N-cadherin in the HME1 (AK + I) cells compared to HME1 (Figure 1A). Based on these observations, we next checked if any changes at the molecular level supported the cytoskeleton rearrangements observed. We screened both the HME1 and HME1 (AK + I) cells for the markers of epithelial-to-mesenchymal transition (EMT) using Hela cells as a positive control, and we also investigated their invasive potential. We detected the significant upregulation (*p* < 0.05) of the mesenchymal markers Zonula occludens (ZO-1), ß-catenin, and N-cadherin and the downregulation of the epithelial marker E-cadherin (*p* < 0.001) (Figure 1B,C) in HME1 (AK + I), which is in contrast to the control HME1 cells. ZO-1 is a major component of adherents and tight junctions, and its downregulation in most cancers has always been associated with cellular motility. However, the dual role of ZO-1 as a pro- and anti-angiogenic molecule has also been appreciated [51].

We next investigated the cellular motility and collagen invasion potential. Metastasis is a key feature of cancer cells and a final step in tumor progression. Invasion is the very first step of cell metastasis. It is a process through which malignant cells detach from the tumor mass, acquire motility, and invade surrounding tissues [52]. Consistent with our previous data, the collagen invasion assay showed a higher invasion rate (*p* < 0.05) after cell exposure to adipokines and inflammatory mediators compared to the HME1 cells cultured in their appropriate medium alone (Figure 1D). The microscopy images revealed that the HME1 (AK + I) cells were denser than the HME1 cells, showing a more aggressive phenotype, especially in the presence of pleomorphic, polygonal, or hyperchromatic cells with cytoplasmic extensions (Figure 1E). Moreover, according to our qRT-PCR results, the matrix metalloproteinase-9 (MMP9) was overexpressed in the HME1 (AK + I) cells compared to HME1 (eight-fold change) (Figure 4B). MMP9 plays an important role in extracellular matrix (ECM) remodeling and is associated with tumor invasion, metastasis, and tumor micro-environment modulation [53,54]. MMP9 overexpression in obesity-exposed HME1 cells can explain increased cell invasiveness and motility.

When taken together, these results indicate that the exposure of HME1 cells to the obesity micro-environment induces their transformation from epithelial to mesenchymal status, acquiring the ability to become invasive.

### 3.2. Obesity Micro-Environment Induces DNA Damage Accumulation in Cultured Epithelial Cells

**Figure 2:**
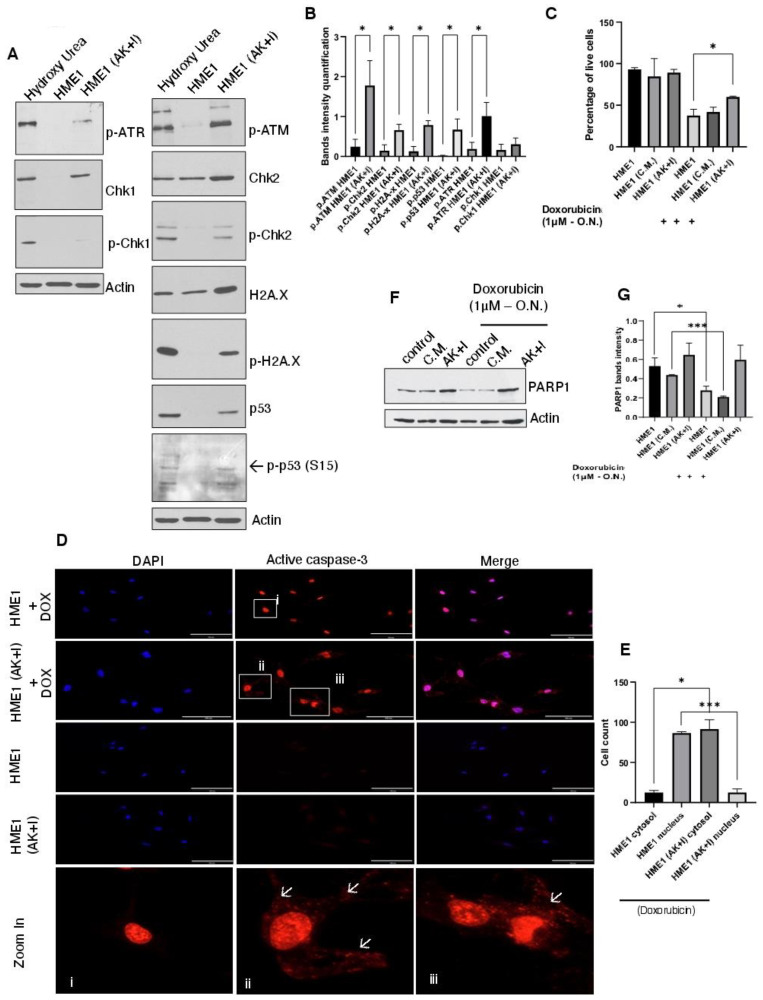
Exposing cells to the obesity micro-environment promotes DNA damage accumulation and chemotherapy resistance. (**A**) Obesity-exposed cells accumulate DNA damage by activating the ATM-Chk2-H2A pathway. HME1 and HME1 (AK + I) cells were lysed and subjected to Western blot analysis for the indicated proteins. Hydroxyurea (1 mM, 48 h), a DNA damage reagent, was used as a positive control, and actin was used as a loading control. (**B**) DNA damage band quantification. Values are represented as mean ± SEM, n = 3 (3 biological × 3 technical). Paired T test; * *p* < 0.05. (**C**) Exposure to the obesity micro-environment promotes cellular resistance to chemotherapy. HME1 and HME1 (AK + I) cells were treated overnight with 1 μM of doxorubicin, and then an annexin V–PI test was performed according to the manufacturer’s recommendations. Values are represented as mean ± SEM, n = 3 (3 biological × 3 technical). Paired T test; * *p* < 0.05. (**D**) Chemotherapy-resistant cells prevent active-Caspase-3 nuclear localization. Active Caspase-3 (cleaved Caspase-3 form) was detected by immunofluorescence. HME1 and HME1 (AK + I) cells were either treated (or not) with 1 μM of doxorubicin before active Caspase-3 analysis. HME1 and HME1 (AK + I) cells were stained for active Caspase-3 (red). Nuclei were stained with DAPI (blue). Scale bar: 100 µm. (i) Zoomed image of HME1 cells; (ii,iii) zoomed image of HME1 (AK + I) cells. Arrows indicate active Caspase-3 localized in the cytosol. (**E**) Active Caspase-3 quantification in cytosol vs. nucleus. Graph with the statistical representation of the number of cells showing active Caspase-3 in the nucleus vs. the number of cells showing active Caspase-3 in the cytosol in both HME1 and HME1 (AK + I) cells treated with doxorubicin. Values are represented as mean ± SEM, n = 3 (3 biological × 3 technical). The count comprised 100 cells for each condition. Scale bar: 200 µm. Paired T test; * *p* < 0.05 and *** *p* < 0.001. (**F**) PARP1 cleavage is compromised under obesity conditions. HME1 and HME1 (AK + I) cells were cultured and treated overnight with 1 μM of doxorubicin. Cells were collected and lysed. Cell extracts were analyzed using Western blotting for PARP1 activation. Actin was used as a loading control. (**G**) PARP1 band quantification. Values are represented as mean ± SEM, n = 3 (3 biological × 3 technical). Paired T test; * *p* < 0.05 and *** *p* < 0.001.

DNA damage accumulation and repair failure are well-defined events in cancer cells. In response to DNA damage and stress, cells activate different signaling networks to either mediate cell cycle arrest and DNA repair or trigger apoptosis. DNA damage response is initiated by activating the ataxia telangiectasia-mutated (ATM) and ATM and Rad3-related (ATR) kinases. Additionally, downstream kinases are activated, such as checkpoint kinases 1 and 2 (Chk1 and Chk2), which are encoded by the CHEK1 and CHEK2 genes, respectively [55]. Both pathways can phosphorylate p53 protein at serine 15 to enhance its transactivating/transcriptional activity [56]. In order to check the presence of DNA damage in the obesity micro-environment-exposed cells, we investigated the activation of these pathways in HME1 and HME1 (AK + I) cells. Contrary to HME1, the HME1 (AK + I) cells showed strong ATM phosphorylation and slight ATR phosphorylation (*p* < 0.05). Interestingly, only the downstream pathway of ATM was activated (Figure 2A,B). Hence, we report both Chk2 phosphorylation and p53 phosphorylation at serine 15, indicating its activation. In addition, we checked histone H2A.X phosphorylation, which is a key DNA damage response component located downstream of Chk2. Upon DNA damage, H2A.X is phosphorylated by ATM, and this is positively associated with the degree of DNA damage [57]. In HME1 (AK + I) cells, histone H2A.X was robustly phosphorylated. When taken together, we conclude that, upon exposure to the obesity micro-environment, HME1 cells exhibit evident DNA damage accumulation that triggers the activation of the ATM-Chk2-H2A.X pathway.

### 3.3. In the Obesity Micro-Environment, Cells Become Resistant to Conventional Chemotherapy

Different mechanisms are involved in acquiring drug resistance in cancer cells, such as drug inactivation, multidrug resistance, apoptosis inhibition, epigenetic changes, drug metabolic reprogramming, changing drug targets, DNA repair enhancement, and target gene amplification [58]. We monitored cellular sensitivity to doxorubicin in HME1 vs. HME1 (AK + I) cells. The cells were cultured in their appropriate media (standard media, with the addition of adipokines and inflammatory mediators, or with the adipocyte-conditioned medium) and then treated overnight with 1µM of doxorubicin (IC50). As is evident from the annexin-FITC results (Figure 2C), nearly 60% of the HME1 cells treated with doxorubicin died compared to the HME1 cells without any treatment. Interestingly, the HME1 cells cultured in the obesity micro-environment were less sensitive to doxorubicin, showing only 30% cell death. This is consistent with the fact that obese individuals develop drug resistance and become less responsive to conventional chemotherapy. The HME1 cells exposed to conditioned media only showed an insignificant increase in cell survival compared to HME1 (AK + I).

**Figure 3:**
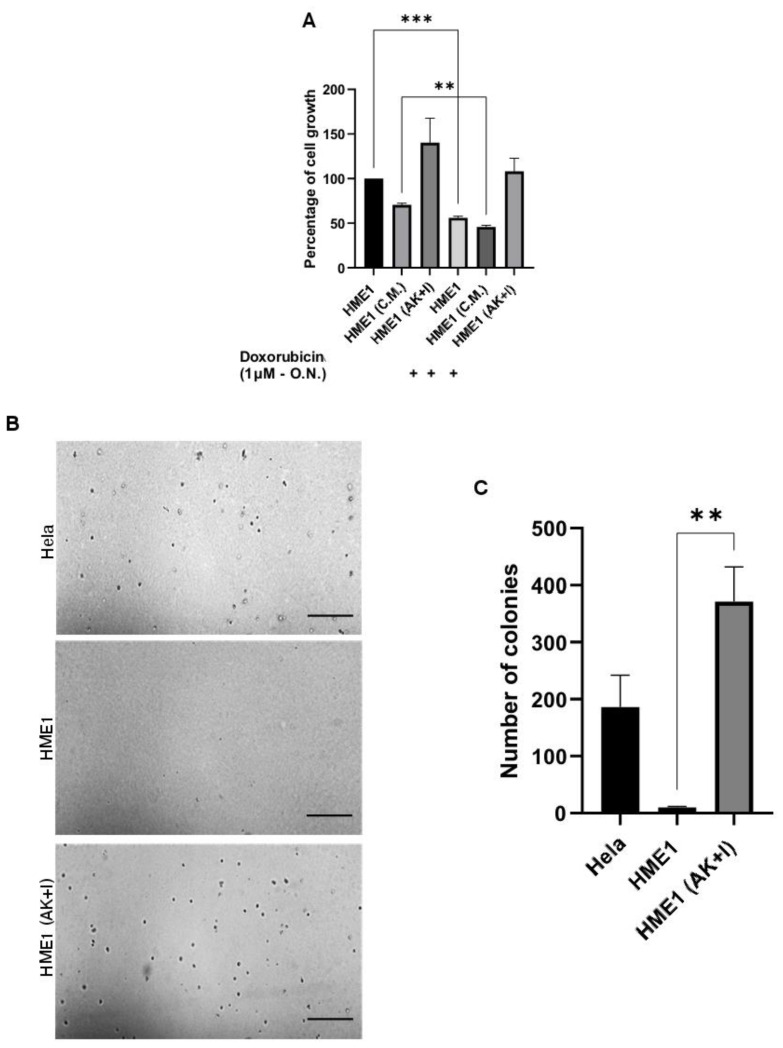
Adipokines and inflammatory mediators promote cellular proliferation. (**A**) Adipokines and inflammatory mediators promote cellular proliferation. The effect of the obesity micro-environment on HME1 cells proliferation was measured using the MTT colorimetric method. Values represent cell density based on the measurement of the optical density at 570 nm using a 96-well plate normalized to the control cells (HME1). HME1 (CM) cells cultured in a conditioned medium collected from pre-adipocytes cells 3T3-F442A. Details about the conditioned medium can be found in Materials and Methods in the cell culture section. Values are represented as mean ± SEM, n = 3 (3 biological × 3 technical). Paired T test; ** *p* < 0.01 and *** *p* < 0.001. (**B**) Obesity micro-environment favors colony formation. We tested the effect of obesity micro-environment on anchorage-independent cell growth/colony formation in soft agar. Single-cell suspensions of Hela, HME1, and HME1 (AK + I) were seeded in soft agar, as described in Materials and Methods. Hela cell line was used as a positive control. Photographs represent microscopic fields of soft agar-grown colonies observed on day 30 after seeding. Scale bar: 1 mm. (**C**) The graph represents the number of colonies formed by the HME1 and HME1 (AK + I) cells. Values represent mean ± SEM, n = 3 (3 biological × 3 technical). Paired T test; ** *p* < 0.01.

Caspase activation is a common signaling cascade in apoptosis. Once activated, through cleaving and activating effector caspases, such as the poly(ADP-ribose)polymerase 1 (PARP1), caspases initiate cell death [59]. We investigated Caspase-3 and PARP-1 activation and degradation upon exposure to chemotherapeutic drugs under the obesity micro-environment. Since the nuclear translocation and accumulation of active Caspase-3 are hallmarks of apoptosis [60,61], we monitored the subcellular localization of Caspase-3. Both the HME1 and HME1 (AK + I) cells were stained for active Caspase-3 in the presence or absence of doxorubicin. In both HME1 and HME1 (AK + I), we did not detect any active Caspase-3 signal. Interestingly, once the cells were treated with doxorubicin, active Caspase-3 was almost fully localized in the nuclei of the HME1 cells, with 91 cells out of 100 total showing active Caspase-3 accumulation in the nucleus, while only nine cells showed active Caspase-3 accumulation in the cytosol. As for the HME1 (AK + I) cells, a significant pool of active Caspase-3 remained in the cytosol, with 85 cells out of 100 showing this phenotype. Interestingly, only 15 cells out of 100 showed active Caspase-3 in the nucleus (Figure 2D,E). These results are in accordance with the MTT assay (Figure 3A), where we showed that HME1 cells treated with doxorubicin exhibit more sensitivity to the treatment (40% of cell death) compared to HME1 cells exposed to the obesity micro-environment where we see a very mild effect (nearly 20% of cell death). We next investigated the caspase substrate PARP-1 under these conditions (Figure 2F,G). Consistent with our earlier findings, the HME1 cells exposed to adipokines and the inflammatory mediators demonstrated less sensitivity to doxorubicin, indicated by less PARP-1 cleavage, which was not the case for the HME1 cells exposed to conditioned media.

### 3.4. Obesity Micro-Environment Promotes Cellular Proliferation and Anchorage-Independent Cell Growth

Unlimited cell proliferation, anchorage-independent growth, and invasiveness are predominant features of cancer cells [62]. Therefore, we tested the proliferation, transformation ability, and clonogenic capacity of the HME1 and HME1 (AK + I) cells. As shown in Figure 3A, the HME1 (AK + I) cells had a higher proliferation rate compared to HME1, where the cells show 40% more proliferation. When compared to the HME1 (AK + I) and HME1 cells, the HME1 cells exposed to adipocyte conditioned media alone without inflammatory mediators showed no increase in the proliferation rate. Figure 3B,C represent the colony-forming ability of the cells. The Hela cells were used as a positive control (186 colonies); as for HME1 cells, they formed 10 colonies (compared to 371) once they were cultured in the obesity micro-environment. This data demonstrates that exposing cells to the obesity micro-environment can increase cell proliferation, ultimately resulting in malignant transformation marked by anchorage-independent cell growth and collagen invasion.

### 3.5. Gene Expression Analysis Uncovered a New Pathway Involved in Breast Cancer Development in Obese Individuals

**Figure 4:**
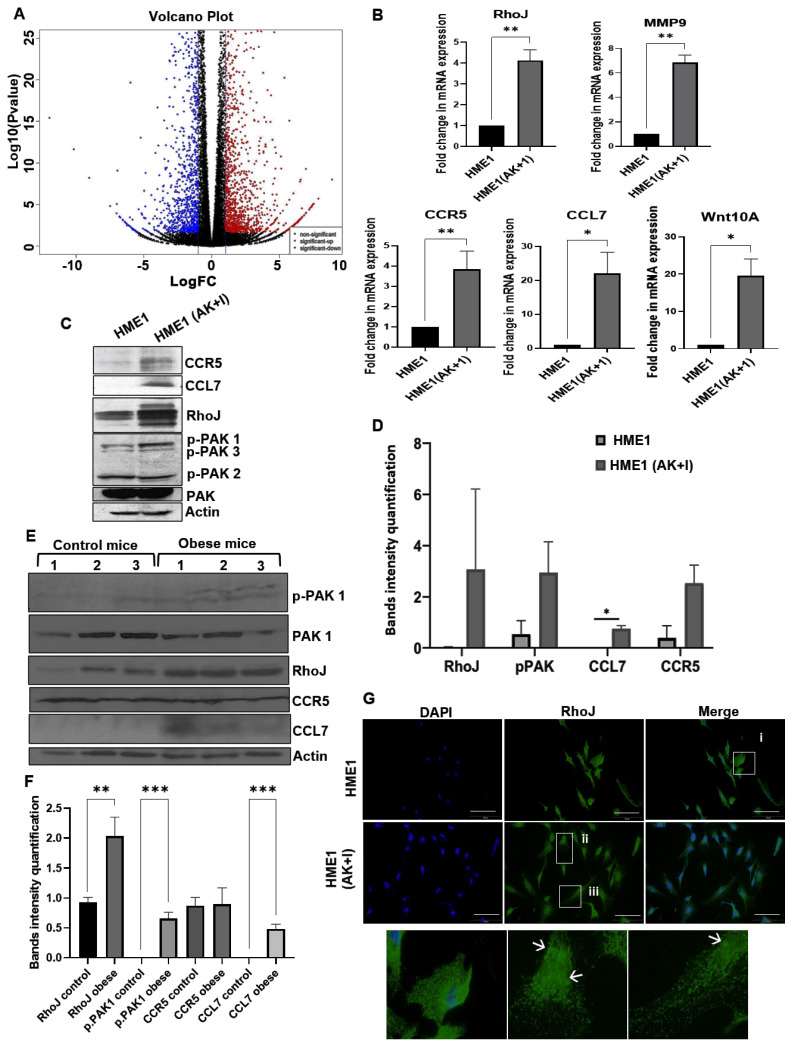
Expression validation of the selected genes after HME1 exposure to obesity micro-environment. (**A**) RNA sequencing. The figure contains a scatter plot for HME1 and HME1 (AK + I) comparison, which displays the LOG2-fold changes and statistical significance of each gene calculated by performing a differential gene expression analysis. Every point in the plot represents a gene. Red points indicate significant upregulated genes; blue points indicate downregulated genes. (**B**) Micro-array validation. Real-time PCR measurement of MMP9, RhoJ, CCL7, CCR5, and Wnt10A expression in HME1 and HME1 (AK + I) cells. Graphs represent the fold change of each gene in both cell lines. Values represent mean ± SEM, n = 3 (3 biological × 3 technical). Paired T test; * *p* < 0.05 and ** *p* < 0.01. (**C**) RhoJ-MMP9 as a potential pathway responsible for epithelial cells transformation in obese individuals. HME1 and HME1 (AK + I) cells were collected and lysed. Total cell extract was analyzed using Western blot for the indicated proteins. Actin was used as a loading control. (**D**) Target protein quantification. Values represent mean ± SEM, n = 3 (3 biological × 3 technical). Paired T test; * *p* < 0.05. (**E**) Pathway validation in vivo. Two groups of mice were used, with three mice per group. One group of mice was given normal food (control diet), and the other group was given high-fat diet food (HFD diet) for nearly 4 months. Every 10 days, the mice were weighed until the HFD diet group mice weight doubled that of the control diet group mice. Mice were sacrificed, and the mammary tissue was collected, lysed, and homogenized. An equal amount of protein was loaded, and antibodies against PAK1/P-PAK1, RhoJ, CCR5, and CCL7 were used. Actin was used as a loading control. Tissues were collected from three different control mice and three different HFD mice. (**F**) In vivo protein expression quantification. Graph representing protein quantification based on band intensity. Values are represented as mean ± SEM, n = 3 (3 biological × 3 technical). Unpaired T test; ** *p* < 0.01 and *** *p* < 0.001. (**G**) RhoJ is activated in the obesity micro-environment. HME1 and HME1 (AK + I) cells were stained for RhoJ (green). Nuclei were stained with DAPI (blue). The top panel represents the HME1 cells; the bottom panel represents the HME1 (AK + I) cells. Scale bar: 100 μm. (i) Zoomed image of HME1 cells; (ii,iii) zoomed images of HME1 (AK + I) cells. Arrows indicate RhoJ activation.

After characterizing the HME1 (AK + I) cells at different levels (both phenotypically and molecularly), and after showing that these cells carry distinct hallmarks of cancerous cells, we next submitted both HME1 and HME1 (AK + I) for transcriptomic analysis using next-generation sequencing (NGS) to define the different target genes and possible pathways involved in cellular transformation and invasion (Figure 4A). The dysregulated genes were classified into different groups according to their cellular functions. Several interesting genes were identified, and a few were selected for validation by qRT-PCR (Figure 4B) (Supplementary Figure S1) based on their expression level, their role in breast cancer development, and the potential connection between them. All five genes (CCL7, CCR5, RhoJ, MMP9, and PAK1) were the top-regulated genes in the HME1 (AK + I) cells based on the NGS results. These results were confirmed by qRT-PCR.

According to the literature and based on our data, we next intend to understand whether CCL7, CCR5, RhoJ and MMP9 are involved in HME1 cellular transformation, and the potential link between them.

#### 3.5.1. CCR5 and CCL7

The pathologic expression of CCR5 has been found in numerous tumors [33,34,63,64,65,66,67,68,69]. In breast cancer, CCR5 is overexpressed compared to normal tissues, and its expression correlates with an increased migratory potential [70]. In mice, CCR5-positive breast cancer cells have an increased ability to form mammospheres and, consequently, form tumors [67]. The expression of the ligands of this receptor is responsible for lymphocytes’ attraction to the tumor micro-environment. CCL7 is one of many chemokines capable of binding and activating the CCR5 receptor [71,72,73]. In our lab, we compared both CCR5 and CCL7 expression levels in HME1 cells when exposed (or not) to the obesity micro-environment (Figure 4B,C). Our results showed CCR5 and CCL7 expression only in the obesity context (a 4-fold change and 15-fold change, respectively), while the HME1 cells cultured in the normal media without any additives showed no expression. We sought to confirm these data in vivo (Figure 4E,F); for this purpose, six female c57Bl/6 mice were divided into two groups of three mice each. One group of control mice was fed a standard diet, whereas the second group was fed a high-fat diet (HFD) to promote obesity. After 14 weeks, the HFD-fed mice weighed nearly twice that of the control group, 45 g to 25 g, respectively (Supplementary Figure S2A,B); the mice were sacrificed, and the mammary gland was collected for protein analysis. CCR5 expression showed no difference between the two groups of mice. Interestingly, CCL7 was only detected in the HFD-fed mice, indicating the potential activation of the CCR5 receptor in obesity conditions only. The difference in CCR5 expression in vitro and in vivo could be related to the difference in CCR5 expression regulation that can be related to genetic factors: factors involved in the activation, signaling, and trafficking of the receptor or to environmental triggers [74]. We assume that, in vivo, despite the expression of the CCR5 receptor in both groups of mice, its activation is associated with the presence of its ligand CCL7, which was observed in obese mice only.

#### 3.5.2. RhoJ and PAK1

Recent studies indicate that the RhoJ regulates cell migration and invasion through altering actin cytoskeletal dynamics in malignant melanoma [33]. Based on these findings, we investigated the expression and activation of RhoJ and PAK1 in both our conditions. Interestingly, in our model, and as was expected, RhoJ expression was upregulated in the HME1 cells exposed to the obesity micro-environment (4-fold change) compared to HME1 cells at both the mRNA and protein levels (Figure 4B,C). Next, we sought to verify the status of RhoJ activation under our experimental conditions. Most Rho GTPase family members contain a hydrophobic lipid tail, promoting the proper subcellular localization of active Rho GTPase to the plasma membrane [33]. Studies have shown that constitutively active RhoJ is specifically localized in the plasma membrane [70]. Moreover, Ackerman et al. have shown that RhoJ distribution and activation indicate GTP loading at or near the plasma membrane [67]. In order to check if RhoJ is not only stabilized in HME1 (AK + I) cells but is also active, we monitored RhoJ localization within the cells by immunofluorescence. As depicted in Figure 4G, contrary to HME1, HME1 (AK + I) showed RhoJ localization in the plasma membrane, indicating its activation. Furthermore, we checked PAK1 phosphorylation status and activation. PAK1 is the direct downstream target of RhoJ. As expected, we observed PAK1 phosphorylation in the HME1 (AK + I) cells, indicating RhoJ activity (Figure 4C). RhoJ accumulation and activation were monitored in vivo as well, where the HFD-fed mice showed the distinct upregulation of RhoJ levels (*p* < 0.01) and PAK1 phosphorylation (*p* < 0.001) compared to the control mice (Figure 4E,F).

### 3.6. Breast Cancer Cell Line Screening

**Figure 5:**
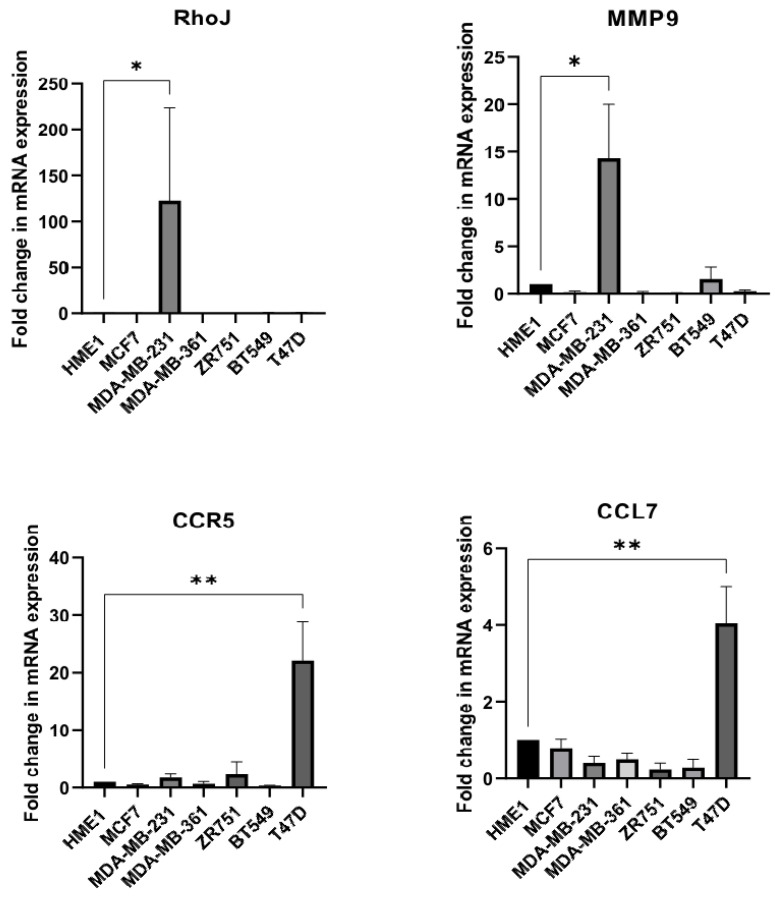
Gene expression screening in a panel of breast cancer cell lines. Normalized relative expression CCR5, CCL7, MMP9, and RhoJ genes in the seven breast cell lines included in this study. Gene expression was determined using Real-Time Quantitative Reverse Transcription PCR (Real-time RT PCR). Each cell line was assayed three times from independent batches of extracted RNA. Graphs represent the fold change of each gene in each cell line. Values represent mean ± SEM, n = 3 (3 biological × 3 technical). Paired T test; * *p* < 0.05 and ** *p* < 0.01.

Finally, we checked whether the pathway we uncovered is a general aspect of breast cancer development or if it is only related to obesity-mediated breast cancer. For this purpose, we investigated CCL7, CCR5, RhoJ, and MMP9 expression in different breast cancer cells: MCF7, MDA-MB-231, MDA-MB-361, ZR751, BT549, and TD47D by using HME1 as a control. Interestingly, both the ligand (CCL7) and its receptor (CCR5) were upregulated in T47D (3-fold and 16-fold change, respectively (*p* < 0.01)), and the upregulation of both RhoJ and MMP9 was observed in the MDA-MB-231 cells (122-fold and 12-fold change, respectively (*p* < 0.05) (Figure 5). Both T47D and MDA-MB-231 are metastatic cell lines, which is consistent with our hypothesis. However, none of the cell lines tested showed the upregulation of all four targets at the same time, which is the case in the HME1 (AK + I) cells.

## 4. Discussion and Conclusions

The association between obesity and cancer is very well-established in the human population. This link has been supported by animal models as well. In our work, we sought to establish the link between obesity and cancer at a molecular level, which can be useful in the future for the prevention of cancer development in obese people and, most importantly, therapeutic intervention.

We adopted a wider perspective whereby we combined the two hallmarks of obesity—tissue dysfunction, marked by a decrease in adiponectin secretion, and chronic inflammation, marked by an increase in the secretion of inflammatory mediators. Our aim was to understand how these two factors engender carcinogenesis at the molecular level. In order to achieve this aim, we cultured HME1 cells, a mammalian mammary epithelial cell line in a special medium to mimic the obesity micro-environment by combining low concentrations of adiponectin and different inflammatory mediators together (leptin, adiponectin, TNF-α, IL-6, and PAI-1). To note, each of these molecules was tested separately, and their role in EMT, cancer cell migration, and invasion has been extensively studied: Leptin [75,76,77], IL-6 [78,79], TNF-α [80,81], PAI-1 [82], and adiponectin [83,84]. To our knowledge, this is the first study where all of these have been combined together with the aim of understanding the role of the obesity micro-environment regarding cancer biogenesis and development (rather than the role of one molecule or another in an isolated setting).

Once exposed to the obesity micro-environment, the HME1 cells showed a drastic rearrangement in their cytoskeleton. The cells acquired a mesenchymal phenotype, accompanied, at the molecular level, by the upregulation of mesenchymal markers. In order to further characterize these transformed cells, we checked different hallmarks of cancerous cells [85]. The obesity micro-environment-exposed cells showed DNA damage accumulation via the activation of the ATM/Chk2 pathway, leading to histone phosphorylation and p53 accumulation and activation. Additionally, the cells showed a higher proliferation rate and acquired anchorage-independent growth and colony formation abilities. Interestingly, these cells also acquired resistance to conventional chemotherapy treatment compared to their matching control. The caspase cleavage of PARP1 occurs in the nucleus under DNA damage signals [86]. We showed a pathological alteration in Caspase-3 activity in obesity conditions, characterized by its localization in the cytosol, accompanied by a decrease in PARP1 cleavage, which, consequently, led to the evasion of apoptotic cell death. After their characterization, both the HME1 and HME1 (AK + I) cells were sent for RNA sequencing. We have chosen different targets from the list according to their role in cancer progression and their potential relationship to each other.

Recent studies have explored the role of RhoJ in melanoma, where RhoJ was found to regulate cell migration and invasion through altering actin cytoskeletal dynamics [35]. Its depletion was shown to be associated with both increased actomyosin contractility and an increased level of active RhoA and phosphorylated light chain (p-MLC). Recent data showed that RhoJ could induce endothelial inflammation when endothelial homeostasis is disrupted. Under physiological conditions, high shear stress downregulated the mRNA and protein levels of RhoJ in endothelial cells, and the siRNA silencing of endogenous RhoJ expression in HUVECs (primary human umbilical vein endothelial cells) downregulated proinflammatory vascular cell adhesion molecule 1 (VCAM-1) and intercellular cell adhesion molecule 1 (ICAM-1) [87]. Furthermore, studies have shown that RhoA depletion enhances CCR5 expression and breast cancer metastasis [88]. In knowing that RhoJ depletion increases RhoA levels and RhoA depletion promotes CCR5 overexpression, we wonder if, in the obesity micro-environment, RhoJ overexpression facilitates the upregulation of CCR5 and, consequently, its ligand CCL7. We hypothesize that the obesity micro-environment leads to RhoJ overexpression, leading to the enhanced expression of CCR5 and CCL7 and increased monocyte recruitment, creating a favorable environment for cell transformation and tumor progression. Further experiments are needed to demonstrate this hypothesis. It was reported that Rho GTPases could modulate the activity of CREB as well, and CCR5 is a well-established transcription target of the cAMP/PKA/CREB pathway. We propose that RhoJ may promote CCR5 and CCL7 overexpression through one or more of these signaling pathways [89,90]; further investigation will be needed to make a clear statement.

Hsiang Ho et al. suggested that RhoJ might play a role in regulating chemoresistance by activating PAK1 in melanoma [91]. Interestingly, PAK1 was found to be upregulated in some breast cancers, and its overexpression was observed in 34 out of 60 breast tumor specimens [92]. PAK1 expression in human breast tumors correlates with tumor grade [93], and it was shown to increase breast cancer cell invasion [68]. According to our results, we proved that HME1 cells exposed to an obesity micro-environment did not respond effectively to conventional chemotherapy compared to HME1 cells, where they showed resistance to apoptosis. Moreover, we have demonstrated the expression of RhoJ and its activation in these cells through PAK1 phosphorylation. The elevated resistance to chemotherapy in HME1 (AK + I) cells suggests that RhoJ, through its direct activation of PAK, might be responsible for cell chemoresistance potential and cellular cytoskeletal rearrangement in the obesity micro-environment, especially considering that PAK1 regulates LIMK, cofilin, and p41-ARC (ARP2/3 complex subunit) [94].

The major limitation of this study was that we could not quantify the total protein content of ATR and ATM. However, we measured the amount of phosphorylation of these proteins, which may be biologically more relevant than the total protein content. 

**Figure 6:**
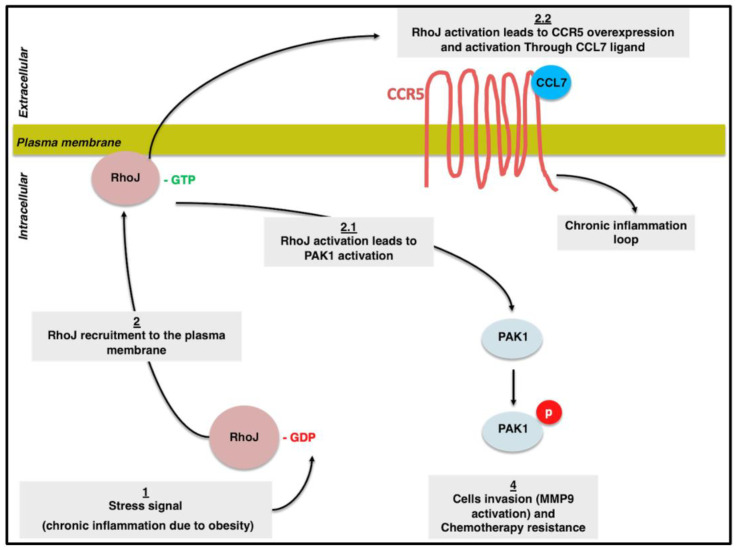
Schematic representation of the different pathways involved in carcinogenesis in obesity conditions. Upon exposure to the obesity micro-environment, we propose that RhoJ is overexpressed and activated, leading to both CCR5 overexpression and activation through CCL7 ligand expression; this creates a closed loop of inflammation and PAK1 phosphorylation and activation, leading to cell chemoresistance and invasion through MMP9 activation.

In conclusion, we propose that in obesity, RhoJ plays a dual role: its upregulation promotes CCR5 and, subsequently, CCL7 overexpression, leading to CCR5-positive monocyte recruitment. This can potentially create a non-stop loop of chronic inflammation, leading to acute inflammation. Consequently, a micro-environment rich in cytokines contributes to cellular transformation and a malignant phenotype. At the same time, during obesity, RhoJ is overexpressed and is constitutively active, which leads to phosphorylation and activation. PAK1, in turn, increases cellular resistance to conventional chemotherapy. We also propose that PAK1 promotes cellular invasion through MMP9 activation. According to a study performed on breast cancer, PAK1 was shown to increase MMP-1 and -3 and downregulate MMP-9 [95]. The induction of each MMP is complex and is regulated differently according to its context. Therefore, we propose that, in the context of obesity, PAK1 could activate MMP-9 (Figure 6).

Our work provides experimental evidence that the obesity micro-environment can transform human mammary epithelial cells, unraveling new mechanisms that involve molecular candidates and potential pathways; this opens new perspectives on the relationship between obesity and cancer. These data support the efforts to prevent cancers via the prevention and treatment of obesity. The pathways and markers identified here help identify potential markers with diagnostic potentials that can be used to evaluate the precancerous phenotype in obesity conditions. Moreover, these pathways and markers can be used as targets for developing new therapies for obesity-related breast cancer.

## Figures and Tables

**Table 1:** Different antibodies used and their dilutions.

Primary Antibodies	Dilutions	Species	References	Catalog Number
E-cadherin	1:1000	Rabbit	Cell signaling, Danvers, MA, USA	3195
N-cadherin	1:1000	Rabbit	Cell signaling, Danvers, MA, USA	13116
ZO-1	1:1000	Rabbit	Cell signaling, Danvers, MA, USA	8193
ß-catenin	1:1000	Rabbit	Cell signaling, Danvers, MA, USA	8480
Check1	1:1000	Rabbit	Cell signaling, Danvers, MA, USA	2360s
Phospho-check1	1:1000	Rabbit	Cell signaling, Danvers, MA, USA	2348s
Check 2	1:1000	Rabbit	Cell signaling, Danvers, MA, USA	6334s
Phospho-check2	1:1000	Rabbit	Cell signaling, Danvers, MA, USA	2197s
H2A X	1:1000	Rabbit	Cell signaling, Danvers, MA, USA	7631s
Phospho-H2A x	1:1000	Rabbit	Cell signaling, Danvers, MA, USA	9718s
Phospho-ATM	1:1000	Rabbit	Cell signaling, Danvers, MA, USA	5883p
Phospho-ATR	1:1000	Rabbit	Cell signaling, Danvers, MA, USA	2853p
p53	1:1000	Mouse	Cell signaling, Danvers, MA, USA	48818s
Phospho-p53 (S15)	1:1000	Rabbit	Cell signaling, Danvers, MA, USA	9286s
CCR5	1:1000	Rabbit	Abcam, Cambridge, UK	Ab65850
CCL7	1:1000	Mouse	Biolegend, San Diego, CA, USA	AF-456-SP
RhoJ	1:250	Mouse	Abnova, Taipei, Taiwan	H00057381-M01
ß-Actin	1:1000	Rabbit	Cell signaling, Danvers, MA, USA	4970s

**Table 2:** List of the different primers used and their sequences.

Primers Sequences (Homo Sapiens)	
Wnt10A forward	GTGCTCCTHTTCTTCCTACTGC
Wnt10A reverse	CCTGGCAATGTTAGGCACACTG
RhoJ forward	TTGCTCGGACTGTATGACACCG
RhoJ reverse	CCTGGACATTGTGGTAAGAGGC
CCR5 forward	TCTCTTCTGGGCTCCCTACAAC
CCR5 reverse	CCAAGAGTCTCTGTCACCTGCA
MMP9 forward	GCCACTACTGTGCCTTTGAGTC
MMP9 reverse	CCCTCAGAGAATCGCCAGTACT
CCL7 forward	ACAGAAGGACCACCAGTAGCCA
CCL7 reverse	GGTGCTTCATAAAGTCCTGGACC
GAPDH forward	GTCTCCTCTGACTTCAACAGCG
GAPDH reverse	ACCACCCTGTTGCTGTAGCCAA
RPL18 forward	GCAGAATCCACGCCAGTACAAG
RPL18 reverse	GCTTGTTGTCCAGACCATTGGC

**Table 3:** List of the different antibodies used for immunofluorescence.

Antibodies	Dilutions	Species	References	Catalog Number
Active Caspase-3	1:500	Rabbit	BD Biosciences, San Jose, CA, USA	559565
Alexa fluorTM 594 goat anti-rabbit IgG (H + L)	1:250	Rabbit	Invitrogen, Waltham, MA, USA	A32740
Alexa fluorTM 488goat anti-mouse IgG (H + L)	1:250	Mouse	Invitrogen, Waltham, MA, USA	A32723

## Data Availability

Data are contained within the article and supplementary materials.

## Supplementary Materials

The following supporting information can be downloaded at: https://www.mdpi.com/article/10.3390/cells13020174/s1.

## References

[1] Collaboration NCDRF (2016). Trends in adult body-mass index in 200 countries from 1975 to 2014: A pooled analysis of 1698 population-based measurement studies with 19.2 million participants. Lancet.

[2] Stone T.W., McPherson M., Gail Darlington L. (2018). Obesity and Cancer: Existing and New Hypotheses for a Causal Connection. EBioMedicine.

[3] Swinburn B., Sacks G., Ravussin E. (2009). Increased food energy supply is more than sufficient to explain the US epidemic of obesity. Am. J. Clin. Nutr..

[4] Cameron A.J., Welborn T.A., Zimmet P.Z., Dunstan D.W., Owen N., Salmon J., Dalton M., Jolley D., Shaw J.E. (2003). Overweight and obesity in Australia: The 1999-2000 Australian Diabetes, Obesity and Lifestyle Study (AusDiab). Med. J. Aust..

[5] Bhaskaran K., Douglas I., Forbes H., dos-Santos-Silva I., Leon D.A., Smeeth L. (2014). Body-mass index and risk of 22 specific cancers: A population-based cohort study of 5.24 million UK adults. Lancet.

[6] Renehan A.G., Tyson M., Egger M., Heller R.F., Zwahlen M. (2008). Body-mass index and incidence of cancer: A systematic review and meta-analysis of prospective observational studies. Lancet.

[7] Bray F., Ferlay J., Soerjomataram I., Siegel R.L., Torre L.A., Jemal A. (2018). Global cancer statistics 2018: GLOBOCAN estimates of incidence and mortality worldwide for 36 cancers in 185 countries. CA Cancer J. Clin..

[8] Wu J., Bostrom P., Sparks L.M., Ye L., Choi J.H., Giang A.H., Khandekar M., Virtanen K.A., Nuutila P., Schaart G. (2012). Beige adipocytes are a distinct type of thermogenic fat cell in mouse and human. Cell.

[9] Longo M., Zatterale F., Naderi J., Parrillo L., Formisano P., Raciti G.A., Beguinot F., Miele C. (2019). Adipose Tissue Dysfunction as Determinant of Obesity-Associated Metabolic Complications. Int. J. Mol. Sci..

[10] Kolb R., Zhang W. (2020). Obesity and Breast Cancer: A Case of Inflamed Adipose Tissue. Cancers.

[11] Bou Malhab L.J., Abdel-Rahman W.M. (2021). Obesity and inflammation: Colorectal cancer engines. Curr. Mol. Pharmacol..

[12] De Pergola G., Silvestris F. (2013). Obesity as a major risk factor for cancer. J. Obes..

[13] Cao H. (2014). Adipocytokines in obesity and metabolic disease. J. Endocrinol..

[14] Romacho T., Valencia I., Ramos-Gonzalez M., Vallejo S., Lopez-Esteban M., Lorenzo O., Cannata P., Romero A., San Hipolito-Luengo A., Gomez-Cerezo J.F. (2020). Visfatin/eNampt induces endothelial dysfunction in vivo: A role for Toll-Like Receptor 4 and NLRP3 inflammasome. Sci. Rep..

[15] Abdalla M.M.I. (2022). Role of visfatin in obesity-induced insulin resistance. World J. Clin. Cases.

[16] Dec P., Poniewierska-Baran A., Modrzejewski A., Pawlik A. (2023). The Role of Omentin-1 in Cancers Development and Progression. Cancers.

[17] Lorincz A.M., Sukumar S. (2006). Molecular links between obesity and breast cancer. Endocr. Relat. Cancer.

[18] Iyengar P., Combs T.P., Shah S.J., Gouon-Evans V., Pollard J.W., Albanese C., Flanagan L., Tenniswood M.P., Guha C., Lisanti M.P. (2003). Adipocyte-secreted factors synergistically promote mammary tumorigenesis through induction of anti-apoptotic transcriptional programs and proto-oncogene stabilization. Oncogene.

[19] Ishikawa M., Kitayama J., Nagawa H. (2004). Enhanced expression of leptin and leptin receptor (OB-R) in human breast cancer. Clin. Cancer Res..

[20] Hotamisligil G.S., Shargill N.S., Spiegelman B.M. (1993). Adipose expression of tumor necrosis factor-alpha: Direct role in obesity-linked insulin resistance. Science.

[21] do Nascimento C.O., Hunter L., Trayhurn P. (2004). Regulation of haptoglobin gene expression in 3T3-L1 adipocytes by cytokines, catecholamines, and PPARgamma. Biochem. Biophys. Res. Commun..

[22] Sindhu S., Thomas R., Shihab P., Sriraman D., Behbehani K., Ahmad R. (2015). Obesity Is a Positive Modulator of IL-6R and IL-6 Expression in the Subcutaneous Adipose Tissue: Significance for Metabolic Inflammation. PLoS ONE.

[23] Carter J.C., Church F.C. (2009). Obesity and breast cancer: The roles of peroxisome proliferator-activated receptor-gamma and plasminogen activator inhibitor-1. PPAR Res..

[24] Wrzeszcz K., Rhone P., Kwiatkowska K., Ruszkowska-Ciastek B. (2023). Hypercoagulability State Combined with Post-Treatment Hypofibrinolysis in Invasive Breast Cancer: A Seven-Year Follow-Up Evaluating Disease-Free and Overall Survival. Life.

[25] Wrzeszcz K., Slomka A., Zarychta E., Rhone P., Ruszkowska-Ciastek B. (2022). Tissue Plasminogen Activator as a Possible Indicator of Breast Cancer Relapse: A Preliminary, Prospective Study. J. Clin. Med..

[26] Bhardwaj P., Brown K.A. (2021). Obese Adipose Tissue as a Driver of Breast Cancer Growth and Development: Update and Emerging Evidence. Front. Oncol..

[27] Larsson S.C., Wolk A. (2007). Overweight, obesity and risk of liver cancer: A meta-analysis of cohort studies. Br. J. Cancer.

[28] Vona-Davis L., Rose D.P. (2007). Adipokines as endocrine, paracrine, and autocrine factors in breast cancer risk and progression. Endocr. Relat. Cancer.

[29] Marino N., German R., Rao X., Simpson E., Liu S., Wan J., Liu Y., Sandusky G., Jacobsen M., Stoval M. (2020). Upregulation of lipid metabolism genes in the breast prior to cancer diagnosis. NPJ Breast Cancer.

[30] Macis D., Guerrieri-Gonzaga A., Gandini S. (2014). Circulating adiponectin and breast cancer risk: A systematic review and meta-analysis. Int. J. Epidemiol..

[31] Bielawski K., Rhone P., Bulsa M., Ruszkowska-Ciastek B. (2020). Pre-Operative Combination of Normal BMI with Elevated YKL-40 and Leptin but Lower Adiponectin Level Is Linked to a Higher Risk of Breast Cancer Relapse: A Report of Four-Year Follow-Up Study. J. Clin. Med..

[32] Aindelis G., Tiptiri-Kourpeti A., Lampri E., Spyridopoulou K., Lamprianidou E., Kotsianidis I., Ypsilantis P., Pappa A., Chlichlia K. (2020). Immune Responses Raised in an Experimental Colon Carcinoma Model Following Oral Administration of Lactobacillus casei. Cancers.

[33] Aaronson S.A. (1991). Growth factors and cancer. Science.

[34] Ackermann K.L., Florke R.R., Reyes S.S., Tader B.R., Hamann M.J. (2016). TCL/RhoJ Plasma Membrane Localization and Nucleotide Exchange Is Coordinately Regulated by Amino Acids within the N Terminus and a Distal Loop Region. J. Biol. Chem..

[35] Ho H., Soto Hopkin A., Kapadia R., Vasudeva P., Schilling J., Ganesan A.K. (2013). RhoJ modulates melanoma invasion by altering actin cytoskeletal dynamics. Pigment. Cell Melanoma Res..

[36] Nishizuka M., Arimoto E., Tsuchiya T., Nishihara T., Imagawa M. (2003). Crucial role of TCL/TC10beta L, a subfamily of Rho GTPase, in adipocyte differentiation. J. Biol. Chem..

[37] Xiao L., Wang J., Li J., Chen X., Xu P., Sun S., He D., Cong Y., Zhai Y. (2015). RORalpha inhibits adipocyte-conditioned medium-induced colorectal cancer cell proliferation and migration and chick embryo chorioallantoic membrane angiopoiesis. Am. J. Physiol. Cell Physiol..

[38] Dai X., Cheng H., Bai Z., Li J. (2017). Breast Cancer Cell Line Classification and Its Relevance with Breast Tumor Subtyping. J. Cancer.

[39] Gelsomino L., Giordano C., Camera G., Sisci D., Marsico S., Campana A., Tarallo R., Rinaldi A., Fuqua S., Leggio A. (2020). Leptin Signaling Contributes to Aromatase Inhibitor Resistant Breast Cancer Cell Growth and Activation of Macrophages. Biomolecules.

[40] Rojas A., Liu G., Coleman I., Nelson P.S., Zhang M., Dash R., Fisher P.B., Plymate S.R., Wu J.D. (2011). IL-6 promotes prostate tumorigenesis and progression through autocrine cross-activation of IGF-IR. Oncogene.

[41] Liu W., Wang H., Bai F., Ding L., Huang Y., Lu C., Chen S., Li C., Yue X., Liang X. (2020). IL-6 promotes metastasis of non-small-cell lung cancer by up-regulating TIM-4 via NF-kappaB. Cell Prolif..

[42] Lyu Y., Xu X., Yun J., Yi J., Li X., Liu X., Ling R., Wang L., Fan J. (2017). [TNF-alpha regulates the proliferation of human breast cancer cells via regulation of ceramide content]. Xi Bao Yu Fen Zi Mian Yi Xue Za Zhi.

[43] Teliga-Czajkowska J., Sienko J., Jalinik K., Derlatka P., Danska-Bidzinska A., Czajkowski K. (2019). Plasminogen Activator Inhibitor Type 1 in Blood at Onset of Chemotherapy Unfavorably Affects Survival in Primary Ovarian Cancer. Adv. Exp. Med. Biol..

[44] Kuri-Harcuch W., Green H. (1978). Adipose conversion of 3T3 cells depends on a serum factor. Proc. Natl. Acad. Sci. USA.

[45] Bhaskaran S., Unnikrishnan A., Ranjit R., Qaisar R., Pharaoh G., Matyi S., Kinter M., Deepa S.S. (2017). A fish oil diet induces mitochondrial uncoupling and mitochondrial unfolded protein response in epididymal white adipose tissue of mice. Free Radic. Biol. Med..

[46] Gurley J.M., Ilkayeva O., Jackson R.M., Griesel B.A., White P., Matsuzaki S., Qaisar R., Van Remmen H., Humphries K.M., Newgard C.B. (2016). Enhanced GLUT4-Dependent Glucose Transport Relieves Nutrient Stress in Obese Mice Through Changes in Lipid and Amino Acid Metabolism. Diabetes.

[47] Holland W.L., Miller R.A., Wang Z.V., Sun K., Barth B.M., Bui H.H., Davis K.E., Bikman B.T., Halberg N., Rutkowski J.M. (2011). Receptor-mediated activation of ceramidase activity initiates the pleiotropic actions of adiponectin. Nat. Med..

[48] Hoda M.R., Keely S.J., Bertelsen L.S., Junger W.G., Dharmasena D., Barrett K.E. (2007). Leptin acts as a mitogenic and antiapoptotic factor for colonic cancer cells. Br. J. Surg..

[49] Dubois V., Delort L., Billard H., Vasson M.P., Caldefie-Chezet F. (2013). Breast cancer and obesity: In vitro interferences between adipokines and proangiogenic features and/or antitumor therapies?. PLoS ONE.

[50] Nair V.A., Valo S., Peltomaki P., Bajbouj K., Abdel-Rahman W.M. (2020). Oncogenic Potential of Bisphenol A and Common Environmental Contaminants in Human Mammary Epithelial Cells. Int. J. Mol. Sci..

[51] Smalley K.S., Brafford P., Haass N.K., Brandner J.M., Brown E., Herlyn M. (2005). Up-regulated expression of zonula occludens protein-1 in human melanoma associates with N-cadherin and contributes to invasion and adhesion. Am. J. Pathol..

[52] Gerashchenko T.S., Novikov N.M., Krakhmal N.V., Zolotaryova S.Y., Zavyalova M.V., Cherdyntseva N.V., Denisov E.V., Perelmuter V.M. (2019). Markers of Cancer Cell Invasion: Are They Good Enough?. J. Clin. Med..

[53] Gialeli C., Theocharis A.D., Karamanos N.K. (2011). Roles of matrix metalloproteinases in cancer progression and their pharmacological targeting. FEBS J..

[54] Mehner C., Hockla A., Miller E., Ran S., Radisky D.C., Radisky E.S. (2014). Tumor cell-produced matrix metalloproteinase 9 (MMP-9) drives malignant progression and metastasis of basal-like triple negative breast cancer. Oncotarget.

[55] Awasthi P., Foiani M., Kumar A. (2016). ATM and ATR signaling at a glance. J. Cell Sci..

[56] Loughery J., Cox M., Smith L.M., Meek D.W. (2014). Critical role for p53-serine 15 phosphorylation in stimulating transactivation at p53-responsive promoters. Nucleic Acids Res..

[57] Choi M., Kipps T., Kurzrock R. (2016). ATM Mutations in Cancer: Therapeutic Implications. Mol. Cancer Ther..

[58] Mansoori B., Mohammadi A., Davudian S., Shirjang S., Baradaran B. (2017). The Different Mechanisms of Cancer Drug Resistance: A Brief Review. Adv. Pharm. Bull..

[59] Chaitanya G.V., Steven A.J., Babu P.P. (2010). PARP-1 cleavage fragments: Signatures of cell-death proteases in neurodegeneration. Cell Commun. Signal.

[60] Prokhorova E.A., Kopeina G.S., Lavrik I.N., Zhivotovsky B. (2018). Apoptosis regulation by subcellular relocation of caspases. Sci. Rep..

[61] Kamada S., Kikkawa U., Tsujimoto Y., Hunter T. (2005). Nuclear translocation of caspase-3 is dependent on its proteolytic activation and recognition of a substrate-like protein(s). J. Biol. Chem..

[62] Menyhart O., Harami-Papp H., Sukumar S., Schafer R., Magnani L., de Barrios O., Gyorffy B. (2016). Guidelines for the selection of functional assays to evaluate the hallmarks of cancer. Biochim. Biophys. Acta.

[63] Achari A.E., Jain S.K. (2017). Adiponectin, a Therapeutic Target for Obesity, Diabetes, and Endothelial Dysfunction. Int. J. Mol. Sci..

[64] Ahirwar A.K., Jain A., Goswami B., Bhatnagar M.K., Bhatacharjee J. (2014). Imbalance between protective (adiponectin) and prothrombotic (Plasminogen Activator Inhibitor-1) adipokines in metabolic syndrome. Diabetes Metab. Syndr..

[65] Alexandre M., Uduman A.K., Minervini S., Raoof A., Shugrue C.A., Akinbiyi E.O., Patel V., Shitia M., Kolodecik T.R., Patton R. (2012). Tobacco carcinogen 4-(methylnitrosamino)-1-(3-pyridyl)-1-butanone initiates and enhances pancreatitis responses. Am. J. Physiol. Gastrointest. Liver Physiol..

[66] Aldinucci D., Lorenzon D., Cattaruzza L., Pinto A., Gloghini A., Carbone A., Colombatti A. (2008). Expression of CCR5 receptors on Reed-Sternberg cells and Hodgkin lymphoma cell lines: Involvement of CCL5/Rantes in tumor cell growth and microenvironmental interactions. Int. J. Cancer.

[67] Abdouh M., Zhou S., Arena V., Arena M., Lazaris A., Onerheim R., Metrakos P., Arena G.O. (2014). Transfer of malignant trait to immortalized human cells following exposure to human cancer serum. J. Exp. Clin. Cancer Res..

[68] Adam L., Vadlamudi R., Mandal M., Chernoff J., Kumar R. (2000). Regulation of microfilament reorganization and invasiveness of breast cancer cells by kinase dead p21-activated kinase-1. J. Biol. Chem..

[69] Al-Shibli S.M., Harun N., Ashour A.E., Mohd Kasmuri M.H.B., Mizan S. (2019). Expression of leptin and leptin receptors in colorectal cancer-an immunohistochemical study. PeerJ.

[70] Abdulkhaleq L.A., Assi M.A., Abdullah R., Zamri-Saad M., Taufiq-Yap Y.H., Hezmee M.N.M. (2018). The crucial roles of inflammatory mediators in inflammation: A review. Vet. World.

[71] Liu Y., Cai Y., Liu L., Wu Y., Xiong X. (2018). Crucial biological functions of CCL7 in cancer. PeerJ.

[72] de Oliveira C.E., Oda J.M., Losi Guembarovski R., de Oliveira K.B., Ariza C.B., Neto J.S., Banin Hirata B.K., Watanabe M.A. (2014). CC chemokine receptor 5: The interface of host immunity and cancer. Dis. Markers.

[73] Fox J.M., Kasprowicz R., Hartley O., Signoret N. (2015). CCR5 susceptibility to ligand-mediated down-modulation differs between human T lymphocytes and myeloid cells. J. Leukoc. Biol..

[74] Barmania F., Pepper M.S. (2013). C-C chemokine receptor type five (CCR5): An emerging target for the control of HIV infection. Appl. Transl. Genom..

[75] Lin T.C., Hsiao M. (2021). Leptin and Cancer: Updated Functional Roles in Carcinogenesis, Therapeutic Niches, and Developments. Int. J. Mol. Sci..

[76] Park K.B., Kim E.Y., Chin H., Yoon D.J., Jun K.H. (2022). Leptin stimulates migration and invasion and maintains cancer stem--like properties in gastric cancer cells. Oncol. Rep..

[77] Sanchez-Jimenez F., Perez-Perez A., de la Cruz-Merino L., Sanchez-Margalet V. (2019). Obesity and Breast Cancer: Role of Leptin. Front. Oncol..

[78] Kumari N., Dwarakanath B.S., Das A., Bhatt A.N. (2016). Role of interleukin-6 in cancer progression and therapeutic resistance. Tumour Biol..

[79] Raskova M., Lacina L., Kejik Z., Venhauerova A., Skalickova M., Kolar M., Jakubek M., Rosel D., Smetana K., Brabek J. (2022). The Role of IL-6 in Cancer Cell Invasiveness and Metastasis-Overview and Therapeutic Opportunities. Cells.

[80] Shrestha R., Bridle K.R., Crawford D.H.G., Jayachandran A. (2020). TNF--alpha--mediated epithelial--to--mesenchymal transition regulates expression of immune checkpoint molecules in hepatocellular carcinoma. Mol. Med. Rep..

[81] Yu L., Mu Y., Sa N., Wang H., Xu W. (2014). Tumor necrosis factor alpha induces epithelial-mesenchymal transition and promotes metastasis via NF-kappaB signaling pathway-mediated TWIST expression in hypopharyngeal cancer. Oncol. Rep..

[82] Xu J., Zhang W., Tang L., Chen W., Guan X. (2018). Epithelial-mesenchymal transition induced PAI-1 is associated with prognosis of triple-negative breast cancer patients. Gene.

[83] Bocian-Jastrzebska A., Malczewska-Herman A., Kos-Kudla B. (2023). Role of Leptin and Adiponectin in Carcinogenesis. Cancers.

[84] Parida S., Siddharth S., Sharma D. (2019). Adiponectin, Obesity, and Cancer: Clash of the Bigwigs in Health and Disease. Int. J. Mol. Sci..

[85] Hanahan D., Weinberg R.A. (2011). Hallmarks of cancer: The next generation. Cell.

[86] Mashimo M., Onishi M., Uno A., Tanimichi A., Nobeyama A., Mori M., Yamada S., Negi S., Bu X., Kato J. (2021). The 89-kDa PARP1 cleavage fragment serves as a cytoplasmic PAR carrier to induce AIF-mediated apoptosis. J. Biol. Chem..

[87] Liu W., Zeng Y., Huang L., Zhang X., Bi L., Fan W., Wu G. (2023). RHOJ as a novel mechanosensitive modulator of endothelial inflammation. Biochem. Biophys. Res. Commun..

[88] Kalpana G., Figy C., Yeung M., Yeung K.C. (2019). Reduced RhoA expression enhances breast cancer metastasis with a concomitant increase in CCR5 and CXCR4 chemokines signaling. Sci. Rep..

[89] Sordella R., Classon M., Hu K.Q., Matheson S.F., Brouns M.R., Fine B., Zhang L., Takami H., Yamada Y., Settleman J. (2002). Modulation of CREB activity by the Rho GTPase regulates cell and organism size during mouse embryonic development. Dev. Cell.

[90] Banerjee A., Pirrone V., Wigdahl B., Nonnemacher M.R. (2011). Transcriptional regulation of the chemokine co-receptor CCR5 by the cAMP/PKA/CREB pathway. Biomed. Pharmacother..

[91] Ho H., Aruri J., Kapadia R., Mehr H., White M.A., Ganesan A.K. (2012). RhoJ regulates melanoma chemoresistance by suppressing pathways that sense DNA damage. Cancer Res..

[92] Balasenthil S., Sahin A.A., Barnes C.J., Wang R.A., Pestell R.G., Vadlamudi R.K., Kumar R. (2004). p21-activated kinase-1 signaling mediates cyclin D1 expression in mammary epithelial and cancer cells. J. Biol. Chem..

[93] Salh B., Marotta A., Wagey R., Sayed M., Pelech S. (2002). Dysregulation of phosphatidylinositol 3-kinase and downstream effectors in human breast cancer. Int. J. Cancer.

[94] Shi T.T., Li G., Xiao H.T. (2016). The Role of RhoJ in Endothelial Cell Biology and Tumor Pathology. Biomed. Res. Int..

[95] Rider L., Oladimeji P., Diakonova M. (2013). PAK1 regulates breast cancer cell invasion through secretion of matrix metalloproteinases in response to prolactin and three-dimensional collagen IV. Mol. Endocrinol..

