# Supplementary material for: Towards Understanding the Development of Breast Cancer: The Role of RhoJ in the Obesity Microenvironment
Source: Cells. 2024 Jan 17;13(2):174. doi: 10.3390/cells13020174 (PMC10814036; doi:10.3390/cells13020174)
Supplement: Supplementary file 1 [file cells-13-00174-s001.zip › cells-2708942-supplementary.pdf]

Supplementary Material

CCKR	
**MMP9**	**3.03373225**
TACR1	3.55882924
PPARG	-2.56274214
CXCL8	2.43832086
MAP2K6	4.26999554
IER3	-2.04589872
KLF4	-2.62024641
PTGS2	1.96082872
CXCL2	3.09473238

Angiogenesis	
GRB7	-3.22948866
KDR	-2.4390147
PDGFD	2.52043163
PRKCG	-4.02093121
**WNT10A**	**8.79415877**
PTPRB	-2.11744944
FZD3	2.3160884
WNT2B	-2.5043281
GNG7	-2.45354002
GNG11	2.10527558

Cytoskeletal	
**RHOJ**	**3.57268381**
PAK6	2.12772342
ACTC1	2.0582063
ACTRT3	2.57750964

Inflammation	
GNG7	-2.45354002
PLCL1	6.48029635
PAK6	2.12772342
CCR3	4.05285136
ACTC1	2.0582063
**CCL7**	**5.64774207**
ACTRT3	2.57750964
CAMK2B	-3.35551385
CXCL8	2.43832086
FPR3	3.26356853
IL6	2.11546865
PTGS2	1.96082872
FPR1	4.85067119
CCRL2	2.89988295
CCR1	2.64908721
C3AR1	3.45056365
CXCR2	-6.365593
ITGA4	-2.07199983
**CCR5**	**5.66479864**

Supplementary Figure S1: Gene panel selection. After genes classification into two different groups, upregulated and downregulated genes, they were analyzed with the online software PANTHER and classified into different pathways. Genes were selected based on their function in carcinogenesis (angiogenesis and cytoskeletal modulation) and inflammation and on their fold change.

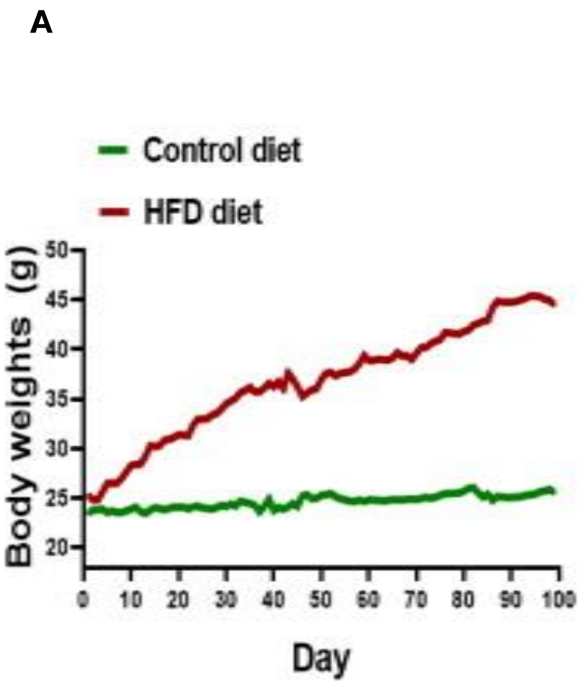

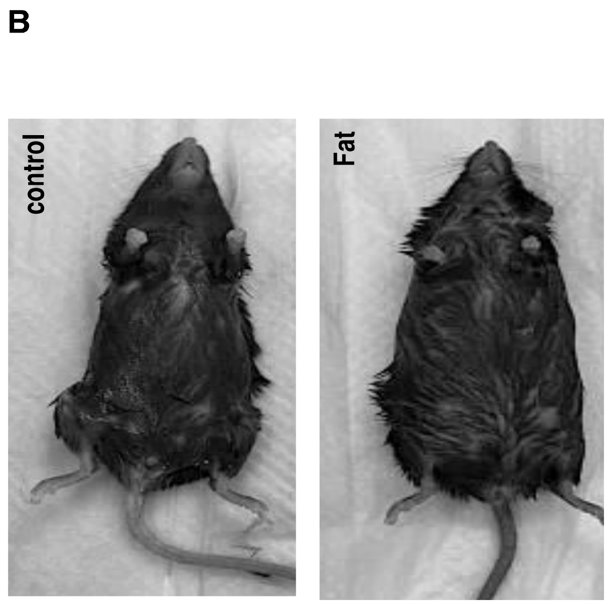

Supplementary Figure S2: (**A**) **Graph representing the body weight evolution between the two mice groups for 100 days.** Two groups of mice were used, with 3 mice per group. One group of mice was given standard chow diet (control group), and the other group was given high-fat diet food (HFD diet) for nearly 4 months. Every 10 days, the mice were weighted until the HFD diet group mice weight twice the control diet group mice. (**B**) Photo representing one mouse of each group.
